# Supplementary material for: Effect of Dietary Supplementation of Bacillus subtilis on Growth Performance, Organ Weight, Digestive Enzyme Activities, and Serum Biochemical Indices in Broiler
Source: Animals (Basel). 2022 Jun 16;12(12):1558. doi: 10.3390/ani12121558 (PMC9219452; doi:10.3390/ani12121558)
Supplement: Supplementary file 1 [file animals-12-01558-s001.zip › animals-1757880-supplementary.pdf]

Supplementary data

Table S1: Composition and Chemical analysis of ingredients (g/kg) of the basal experimental diets (%, as-fed basis).

Ingredients (g/kg)	Starter (1-21day)	Finisher (21-42 day)
Corn	58	63
Soybean (44 % CP)[a]	28.20	24.90
Gluten (60% CP)[b]	7	6
Di calcium phosphate	1.80	1.20
Limestone	1.30	1.30
soybean oil	2	2
Salt	0.30	0.30
L-Lysine HCl	0.20	0.20
DL-Methionine	0.20	0.10
Premix[1]	1	1
Total	100	100
Calculated composition		
Energy (Kcal/kg)[d]	3012.26	3068.97
Crude protein (%)	21.99	20.28
Calcium (%)	0.98	0.84
Total phosphor (%)	0.72	0.59
Methionine (%)	0.59	0.47
Methionine + cysteine (%)	0.95	0.80
Lysine (%)	1.14	1.06
Total	100	100

[a,b]Crude protein

[c]Premix: provided the following per kilogram of diet: 13 000 IU of vitamin A; 1300 IU of vitamin D; 65 IU of vitamin E; 3.4 mg of menadione; 37 mg of pantothenic acide ; 6.6 mg of riboflavin; 3.7 mg of folic acid; 39 mg of Niacin; 1.0 mg of Thiamine; 4.3 mg of Vitamin B6; 0.23 mg Biotin; 0.075 mg of Vitamin B12: 43 mg of choline chloride.

170 mg of Znic; 140 mg of iron; 34 mg of manganese; 16 mg of copper; 0.29 mg of iodine; 0.29 mg of selenium.

[d]Metabolizable energy.

Table S2: The information of all kits used to measure blood biochemical in serum chicken

Total Protein	Total Protein Assay Kit	A045-4-1
Albumin	Albumin assay kit	A110-1-1
Triglyceride	Triglyceride assay kit	A110-1-1
Total cholesterol	Total cholesterol assay kit	A111-1-1
HDL	High-density lipoprotein Assay Kit	A112-1-1
LDL	Low-density lipoprotein Assay Kit	A113-1-1
VLDL	Very low-density lipoprotein Assay Kit	H249
ALP	alkaline phosphatase Assay Kit	A059-3-1
Creatine	Creatine Assay Kit	A032-1-1
Uric acid	Uric acid Assay Kit	C012-2-1
ALT	Alanine aminotransferase Assay Kit	C009-2-1
AST	Aspartate aminotransferase Assay Kit	C010-1-1
Pepsin (U/ml)	Pepsin assay kit	A080-1-1
Lipase (U/l)	Lipase assay kit	A054-1-1
Amylase(U/dl)	α-Amylase Assay Kit	C016-1-1

All commercial kits purchased from Nanjing Jiancheng Bioengineering Institute, Nanjing, China (*http://www.njjcbio.com/*).
